# Kinetic control over the chiral-selectivity in the formation of organometallic polymers on a Ag(110) surface

**DOI:** 10.1038/s42004-024-01137-y

**Published:** 2024-03-05

**Authors:** R. S. Koen Houtsma, Floris van Nyendaal, Meike Stöhr

**Affiliations:** Zernike Institute for Advanced Materials, University of Groningen, Nijenborgh 4, 9747AG Groningen, The Netherlands

**Keywords:** Molecular self-assembly, Synthesis and processing, Surface assembly, Organometallic chemistry

## Abstract

Methods to control chiral-selectivity in molecular reactions through external inputs are of importance, both from a fundamental and technological point of view. Here, the self-assembly of prochiral 6,12-dibromochrysene monomers on Ag(110) is studied using scanning tunneling microscopy. Deposition of the monomers on a substrate held at room temperature leads to the formation of 1D achiral organometallic polymers. When the monomers are instead deposited on a substrate held at 373 K, homochiral organometallic polymers consisting of either the left- or right-handed enantiomer are formed. Post-deposition annealing of room temperature deposited samples at >373 K does not transform the achiral 1D organometallic polymers into homochiral ones and thus, does not yield the same final structure as if depositing onto a substrate held at the same elevated temperature. Furthermore, annealing promotes neither the formation of 1D covalently-coupled polymers nor the formation of graphene nanoribbons. Our results identify substrate temperature as an important factor in on-surface chiral synthesis, thereby demonstrating the importance of considering kinetic effects and the decisive role they can play in structure formation.

## Abstract (web summary)

The effective control of chirality on surfaces is crucial for applications such as enantioselective heterogeneous catalysis and nonlinear optics. Here, the authors study the on-surface synthesis of organometallic polymers and their chiral expression on Ag(110), demonstrating that kinetic effects play an important role in determining polymer chirality.

## Introduction

Chirality is a topic of fundamental importance to many fields of science, including biology, chemistry, and physics^1–7^. The surface science toolkit has been used in previous studies to investigate various on-surface chiral systems and multiple chiral phenomena have been reported, such as chiral amplification, organizational chirality, chirality transfer, chiral switching, chiral surfaces, and chiral-selective synthesis^8–19^. In particular, scanning tunneling microscopy (STM) allows direct, real-space observation of on-surface chiral systems and thus, is a powerful tool for chirality studies^20^. In addition to the fundamental interest in chirality, chiral systems have also been proposed as candidates for future applications, such as in nonlinear optics or liquid-crystal displays^21–24^. One important aspect for future applications is controlling chirality on surfaces since this constitutes the most fundamental level of understanding of the involved processes and emerging properties. Chiral-selective reactions have been used to synthesize various polymers on surfaces in the past and various strategies have been employed to achieve chiral-selectivity. These strategies mostly focus on the molecular structure, for instance by introducing bulky groups causing steric hindrance, to enable certain couplings over others^13,25,26^. On the other hand, also the type of inorganic surface may lead to chiral-selectivity^18,20,27^. In essence, these strategies focus on changing some variables of the chosen molecule substrate system in order to affect chiral-selectivity. However, in addition to these internal factors of the chosen system, external factors enabling chiral-selectivity in on-surface reactions have been rarely studied^28^. Nevertheless, these external factors are important, as they can be used to tune the chirality independent of substrate or molecule choice.

**Fig. 1:**
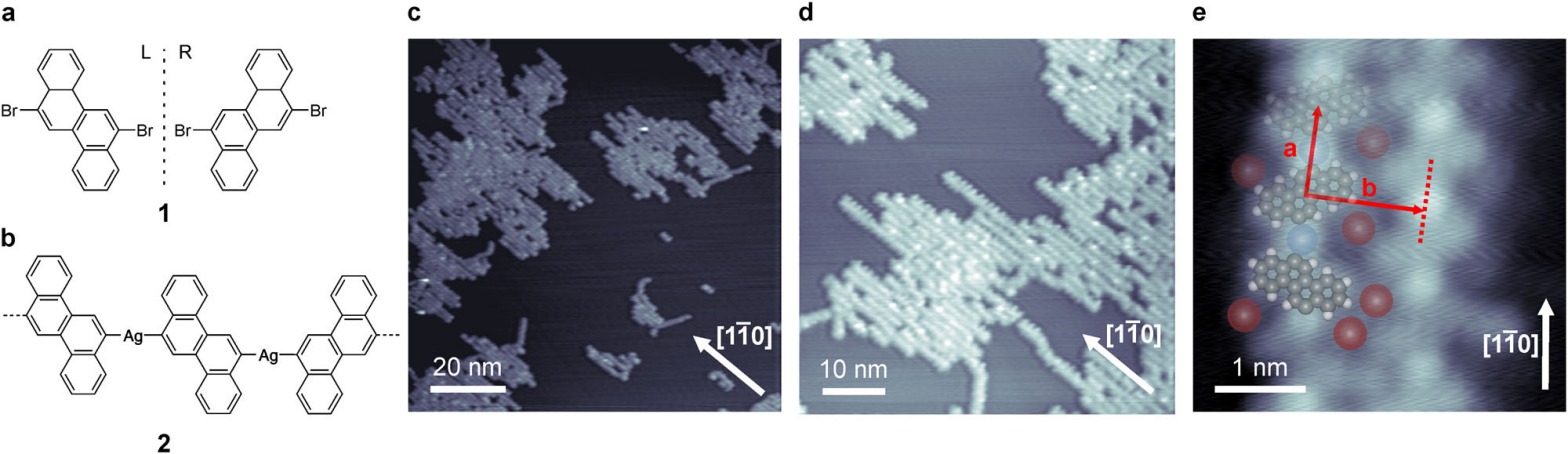
Structure of the organometallic polymers formed at room temperature. **a** Chemical structure of prochiral 6,12-dibromochrysene **1** showing both the left- and right-handed enantiomers which are available on the surface. **b** Schematic of the organometallic polymers **2** formed upon deposition of monomer 1 on Ag(110) held at RT. **c**, **d** Overview STM images of the organometallic polymers **2** formed on Ag(110) upon deposition of **1** onto Ag(110) held at RT. **e** Close-up STM image of two organometallic polymers at an edge of a molecular island with an overlaid molecular model. Deposition was done onto a Ag substrate kept at RT. Scanning parameters: (**c**) −1.9 V, 10 pA, (**d**) −1.9 V, 5 pA, (**e**) 0.5 V, 100 pA. Color code: Br, red; Ag, blue; C, gray; H, white.

In a previous study, prochiral 6,12-dibromochrysene (**1**, Fig. 1a), which is achiral in gas- or liquid phase but becomes chiral once adsorbed on a surface due to the reduction in dimensionality, was shown to form achiral narrow chevron-like graphene nanoribbons (GNRs) on Au(111). On the other hand, chiral 1D organometallic polymers were obtained upon deposition of **1** on Cu(111) held at room temperature (RT). The organometallic polymers could not be converted into GNRs^18^. Thus, monomer **1** has the possibility of forming both GNRs and organometallic polymers on surfaces. Among others, aligning GNRs and polymers is of significant interest for the use in future applications, as aligned structures have been shown to provide better device performance^29–31^. The Ag(110) surface has a row-like geometry since the atoms are more densely packed in the $[1\bar{1}0]$-direction compared to the [001]-direction. This may induce a 1D templating effect and gives the tantalizing possibility to form aligned GNRs on a relatively simple surface^32^. On the other hand, Ag adatoms are already abundantly available at RT on the Ag(110) surface and there exists also the possibility of 1D organometallic polymer development guided by the anisotropic Ag surface, providing further control over chiral-selectivity.

Herein we report the temperature-dependent chiral-selective formation of organometallic polymers from monomer **1** on Ag(110) by means of STM. Upon deposition of monomer **1** on a substrate held at RT, heterochiral 1D organometallic polymers (which consist of a mixture of both enantiomers) were formed on the surface, whereas upon deposition on a substrate held at 373 K homochiral 1D organometallic polymers were formed which further assembled into 2D homochiral networks. We observed that annealing the heterochiral 1D organometallic polymers did not lead to the formation of the homochiral 1D organometallic polymers, i.e. the heterochiral 1D polymers present a kinetically trapped state. Thus, the on-surface reaction, involving the splitting off of the Br atoms of **1** and the subsequent metal-ligand bonding to Ag adatoms, is chiral-selective only when the substrate temperature during deposition is sufficient to prevent kinetic trapping. Chiral-selective synthesis of 1D organometallic polymers based on the surface temperature during deposition has to the best of our knowledge not been reported thus far. We further attempted to transform the organometallic polymers into 1D covalently linked polymers or GNRs. However, the energy barrier to break the metal-ligand bonding between Ag adatoms and **1** for removing the coordinating Ag adatoms in order to covalently couple monomers **1** could not be overcome by any temperature treatment, i.e. neither 1D covalently linked polymers nor GNRs could be synthesized. Our results demonstrate that chiral-selective synthesis may be achieved through reaction kinetics, where in the case of monomer **1** on Ag(110) thermodynamic control leads to the formation of chiral structures, whereas kinetic control leads to achiral structures. Our work shows that the initial kinetic factors can be a crucial factor in determining the chirality of the resultant structure.

## Results and discussion

Deposition of monomer **1** on Ag(110) held at RT led to the formation of molecular islands (Figs. 1c, d and S1 in the supplementary information). Upon closer inspection it becomes clear that the islands consist of parallel arranged 1D molecular chains. The molecular chains turned out to be organometallic polymers, formed with native Ag adatoms^33–35^, which were aligned along the $[1\bar{1}0]$ direction of the Ag(110) surface, indicating that the anisotropic surface geometry had a templating effect for the arrangement of the polymers. The polymers themselves appear as a pattern of alternating stripes and bright protrusions along their longitudinal axes, which we assign to monomer **1** and (native) Ag adatoms, respectively (Fig. 1e). Close-up STM images without overlaid tentative molecular models are available in Figs. S2 and S3. The polymers that constitute the island are relatively short and single polymers do not span the entire length of an island. The polymers appear to preferentially adsorb near surface steps. Upon adsorption of **1** on the Ag(110) surface, the Br atoms get split off from monomer **1** and Ag-coordinated organometallic polymers **2** (Fig. 1b) form. The Ag adatoms originate from the substrate and are already available at RT. A similar (partial) debromination on Ag(110) at room temperature was reported for Br-functionalized thiophene and tetracene derivatives^36,37^. This is similar to the case on the more reactive Cu(111) surface^18,38–40^, but in stark contrast with the case of monomer **1** on Au(111), where an annealing step was required to induce C-Br bond cleavage. However, whereas on Cu(111), the organometallic polymers are homochiral, i.e. formed by only a single type of enantiomer, the polymers formed on Ag(110) are formed by a racemic mixture of both monomer **1** enantiomers, which are not necessarily arranged in an alternating manner. The monomer-to-monomer distance (measured from the center of one monomer to the next along the polymer direction, **a** in Fig. 1e) for the organometallic polymers **2** was found to be 0.97 ± 0.05 nm and the polymers are spaced 1.27 ± 0.05 nm side-to-side (measured perpendicular to polymer direction, **b** in Fig. 1e). This monomer-to-monomer distance rules out the possibility of structure **2** being formed through covalent coupling^18^. Instead, it supports the formation of metal-ligand bonding with a length of 4.7 ± 0.1 Å for the C-Ag-C bond, in good agreement with typical metal-ligand bond lengths^41^. The outside of the organometallic polymers **2** is decorated by dim protrusions whose contrast depends on the applied bias voltage (Fig. S4), which we tentatively assign as Br atoms left on the surface after C-Br bond cleavage took place^40,42,43^. The Br in between the parallel aligned organometallic polymers is suggested to help the formation of the 2D molecular islands through developing Br···H hydrogen bonds^44–46^.

**Fig. 2:**
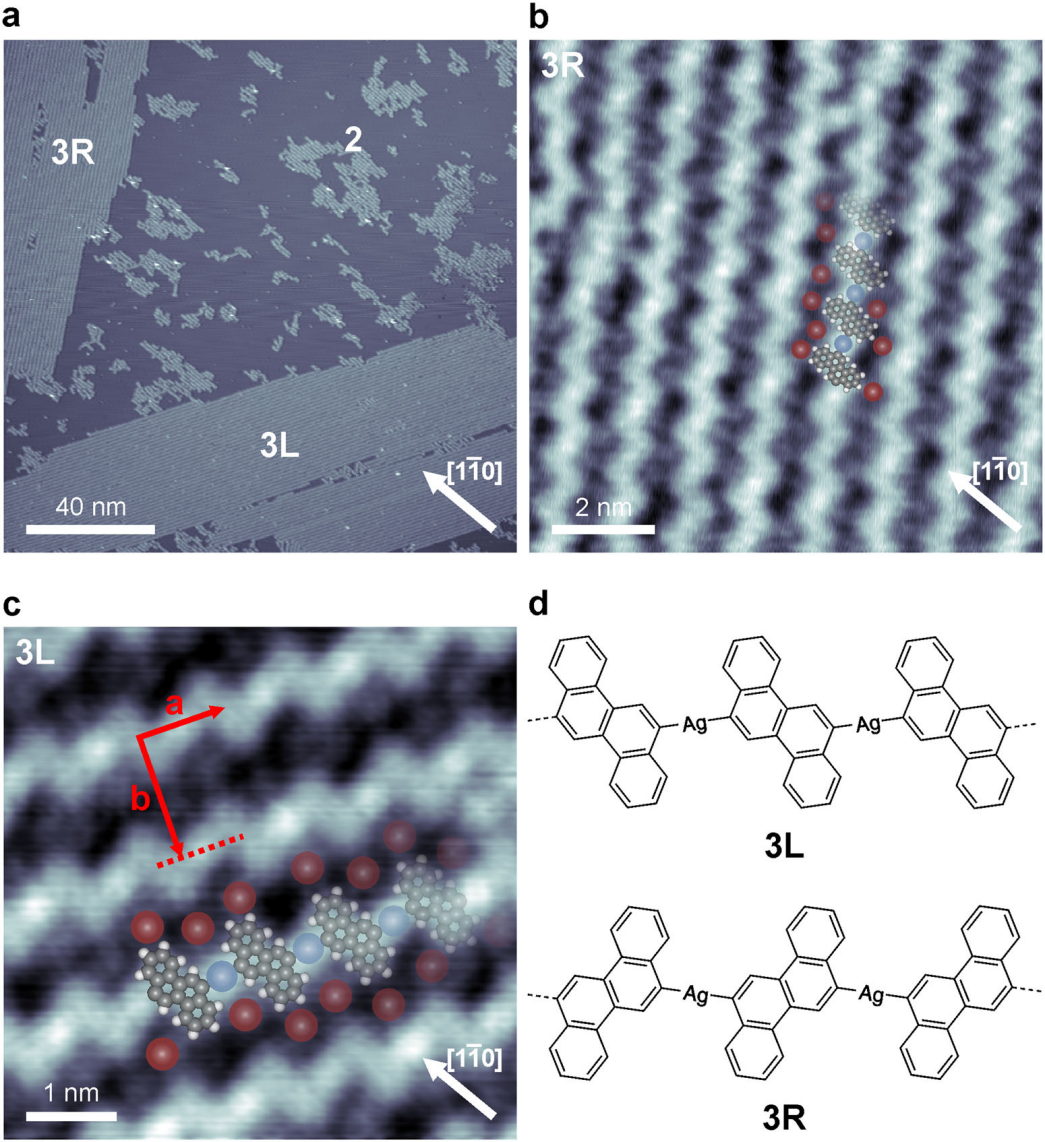
Structure of the organometallic polymers formed upon deposition on Ag(110) held at 373 K. **a** Overview STM image showing organometallic polymers **2**, **3L**, and **3R**. Both 3L and 3R are arranged in extended 2D islands. **b**, **c** Close-up STM images showing the organometallic polymers **3L** and **3R**, respectively with overlaid molecular models, Ag adatoms (blue) and split off Br atoms (red). **d** Schematic of both 1D homochiral organometallic polymers formed upon deposition of monomer **1** on Ag(110) held at 373 K. **3L** consists of only left-handed enantiomers and **3R** of only right-handed ones. Scanning parameters: (**a**) 1.0 V, 30 pA, (**b**) 1.0 V, 100 pA, (**c**) −1.7 V, 500 pA. Color code: Br, red; Ag, blue; C, gray; H, white.

Upon deposition of monomer **1** on Ag(110) held at 373 K three molecular arrangements were observed with STM: arrangement **2**, which was also present upon deposition on a sample held at RT, and two new arrangements labeled **3L** and **3R** (Fig. 2a). The molecular islands of **3L** and **3R** are markedly larger and have better long-range order than those of arrangement **2**. Close-up STM images of **3L** and **3R** arrangements reveal that the large islands are formed by close-packed 1D organometallic polymers (Figs. 2b, c). The polymers once again are made up from alternating stripes and bright protrusions which we assign as monomer **1** and (native) Ag adatoms, respectively. However, in contrast to organometallic polymer **2**, organometallic polymers **3L** and **3R** are exclusively composed of either only left- or right-handed enantiomers. Thus, the organometallic polymers **3L** and **3R** are chiral. Respective tentative molecular models are presented in Fig. 2d. Similar to what was observed for **2**, the homochiral polymers are decorated at their edges with dim protrusions which we again tentatively assign to Br atoms, which may enable the formation of 2D molecular islands through Br···H hydrogen bonds^44–46^. Moreover, the 2D molecular islands are assembled from either **3L** or **3R** polymers which makes the 2D islands homochiral. That means the chirality of the monomers is transferred to the polymers which exist in either the **3L** or **3R** form and which in turn transfer their chirality to the molecular islands. Such a chiral segregation when starting with a racemic mixture has been previously reported for both different chiral and prochiral molecules^18–20,47–49^. The monomer-to-monomer distance (measured analogously as for structure **2,** **a** in Fig. 2c) was determined to 0.96 ± 0.04 nm, again ruling out the possibility of the polymers being formed through covalent coupling^18^. Interestingly, the side-to-side spacing (**b** in Fig. 2c) of 1.35 ± 0.05 nm for the homochiral organometallic polymers **3L**/**3R** is slightly larger than that of the heterochiral organometallic polymer **2**. We hypothesize that this disparity in side-to-side spacing arises from the difference how the polymers adsorb with respect to the principal directions of the Ag(110) substrate. Whereas the organometallic polymer **2** preferentially aligned along the $[1\bar{1}0]$ direction of the substrate, polymers **3L** and **3R** aligned in such a way that the long axes of monomers **1** are parallel to the $[1\bar{1}0]$ direction (Figs. 2b, c and S5). Reorientation (through rotation) of molecules **1** involves overcoming a potential barrier which may be at room temperature due to its too large value not possible. In comparison to an Ag(111) surface, this potential barrier is most likely larger on Ag(110) due to the present anisotropy^50^.

**Fig. 3:**
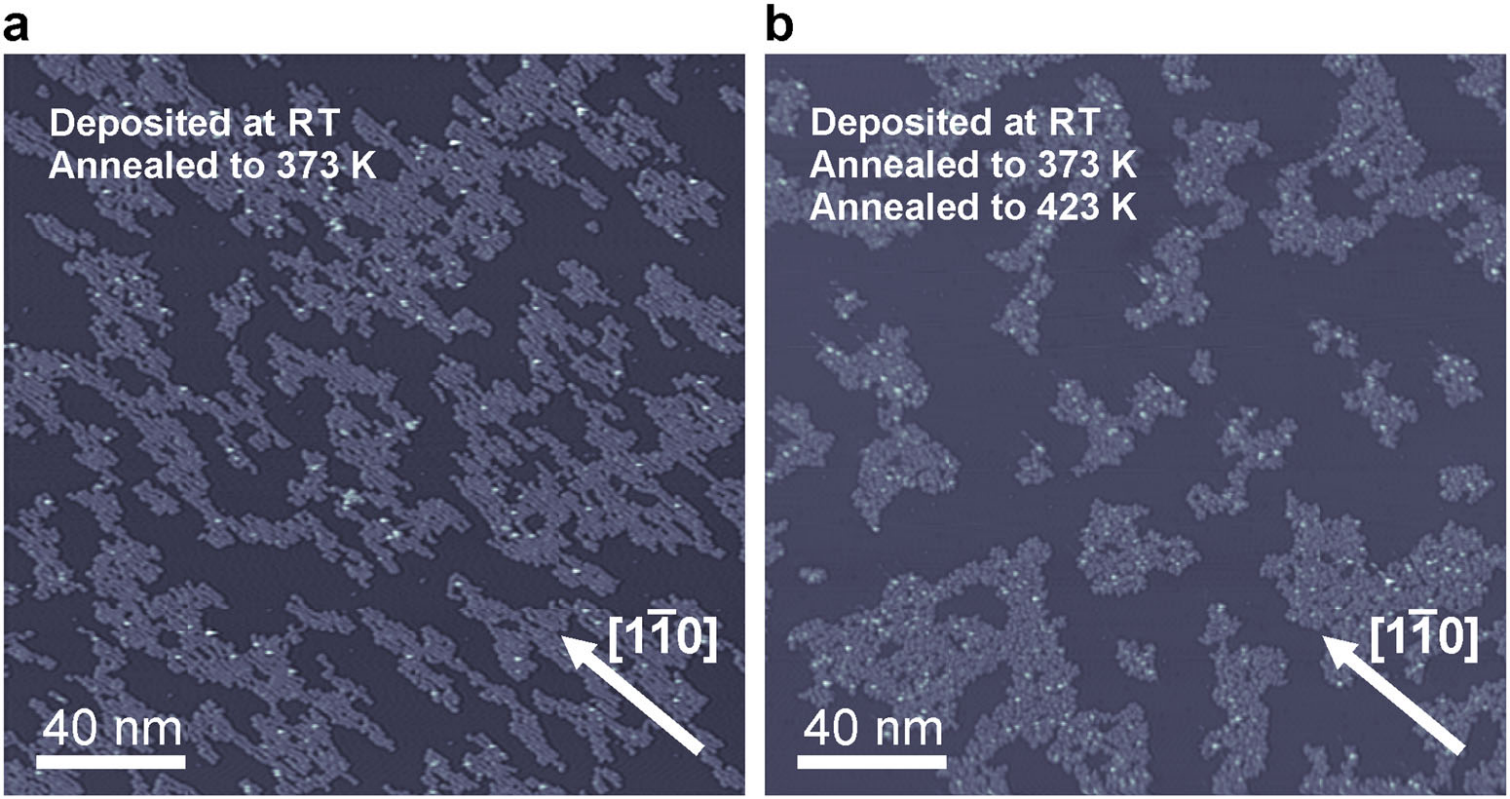
Structure of the samples upon heat treatment. **a** Overview STM image showing the result of annealing a sample deposited at RT and annealed at 373 K. **b** Overview STM image of the same sample as in **a**, but further annealed at 423 K. Scanning parameters: (**a**) −1.9 V, 50 pA, (**b**) −1.9 V, 5 pA.

To investigate whether arrangement **2** could be transformed into arrangement **3L**/**3R** as well as whether any of these structures can be transformed into 1D covalently linked polymers or GNRs as was previously reported for similar organometallic polymers^51,52^, monomer **1** was deposited on Ag(110) at room temperature and subsequently annealed at 373 K. The outcome of such a sample preparation is shown in Fig. 3a. Molecular islands were formed that appear aligned with the $\left[1\bar{1}0\right]$ direction of the substrate and which consist of heterochiral polymers **2**. Although upon annealing the structure of the molecular islands changed, the length of the individual polymers did not seem to be affected (Fig. S6). The homochiral polymers **3L** and **3R** were not observed on the sample. The sample was subsequently further annealed at 423 K (Fig. 3b). Disordered molecular islands were detected and apparently, annealing at these temperatures cannot transform the heterochiral Ag-coordinated polymer **2** into homochiral organometallic polymers **3L**/**3R**. Furthermore, annealing at even higher temperatures (up to 523 K) led to similar results (Fig. S7). Thus, polymer **2** presents a kinetically trapped state which can neither be transformed into **3L**/**3R** nor into covalently linked polymers. Additionally, the molecular coverage decreased upon annealing, indicating a partial molecular desorption and/or decomposition. We further investigated whether organometallic polymers **3L** and **3R** could be transformed into covalent polymers through annealing, which turned out to be impossible as well. These results and their discussion are presented in the Supporting Information (Fig. S8 and Supplementary Note 1).

The formation of the homochiral organometallic polymers **3L** and **3R** upon adsorption on Ag(110) held at 373 K could be attributed to two possible origins: (i) the higher mobility of the monomers adsorbed on the substrate held at 373 K allows for increased diffusion compared to having the Ag substrate at RT. Specifically, the Ag(110) has an anisotropic potential energy landscape, which makes diffusion of Ag atoms (and likely DBCh monomers) more likely along the densely packed rows of the surface compared to across these rows^53^. At elevated temperatures, diffusion across the densely packed rows becomes possible and this enables the sorting of the enantiomers, the chiral separation of the racemic mixture and the subsequent formation of homochiral organometallic polymers can proceed. (ii) The other alternative is that the increased energy per monomer **1** could enable the enantiomers to switch their on-surface chirality through flipping on the surface or through the rotation of a single group within a molecule^3,54^. Chirality switching has been reported for small molecules, *e.g*. for disubstituted benzene molecules^12,55^. Nevertheless, we evaluate mechanism (ii) as unlikely, because flipping of the entire monomer **1**, which is relatively rigid due to its π-conjugation, would be required. In addition, mechanism (ii) would also allow transformation of arrangement **2** into **3L/3R**, which was not observed. Therefore, the formation of arrangements **3L** and **3R** is attributed to mechanism (i), the higher mobility of the monomers on the warm substrate. As a result, the temperature-dependent chiral-selectivity is enabled by the higher mobility of the monomers.

## Conclusion

Upon deposition of the prochiral monomer **1** on Ag(110) held at RT, 1D heterochiral organometallic polymers **2** formed which were found to be assembled in 2D domains through attractive Br···H interactions between the split-off Br atoms and the H atoms of **1**. On the other hand, when monomer **1** was deposited on a sample held at 373 K, 1D homochiral organometallic polymers **3L** and **3R** formed. Again, they assembled into islands through Br···H interactions. Importantly, spontaneous chiral resolution happened, i.e. the 2D islands consisted of either **3L** or **3R** polymers making the islands homochiral. Surprisingly, polymers **2** could not be transformed via post-deposition annealing into polymers **3L**/**3R**. Thus, the synthesis of the organometallic polymers exhibits chiral-selectivity with the sample temperature during deposition as the decisive factor. Furthermore, the organometallic polymers **2,** **3L** and **3R** could not be transformed into covalently coupled polymers. We conclude that polymer **2** is in a kinetically trapped state.

Our work contributes an alternative pathway for enantioselective synthesis on the example of the synthesis of 1D organometallic polymers on a Ag surface with control over the temperature as the important factor.

## Methods

All experiments and sample preparation were conducted under ultra-high vacuum conditions (base pressure: <5 × 10^−10^ mbar). Room temperature in this ultra-high vacuum system amounted to the range between 293 K and 298 K. As substrate a Ag(110) single crystal was used which was cleaned by repeated cycles of Ar^+^ ion sputtering and annealing. The substrate directions were determined through atomically resolved STM images. Commercially available 6,12-dibromochrysene (Sigma-Aldrich) was thermally evaporated at 443 K from a Knudsen cell-type evaporator (Omnivac). Post-deposition annealing was performed through resistive heating. STM imaging was performed using a commercial low temperature STM (Scienta Omicron) operated at 78 K. For all images the STM was operated in constant current mode using mechanically cut PtIr tips. STM data were processed using the WSxM software package^56^.

### Supplementary information

Supplementary Information

## Data Availability

Data supporting the conclusions of this manuscript are available from the corresponding author upon reasonable request.
