# Supplementary material for: Kinetic control over the chiral-selectivity in the formation of organometallic polymers on a Ag(110) surface
Source: Commun Chem. 2024 Mar 5;7:51. doi: 10.1038/s42004-024-01137-y (PMC10914819; doi:10.1038/s42004-024-01137-y)
Supplement: Supplementary file 1 — Supplementary Information [file 42004_2024_1137_MOESM1_ESM.pdf]

Supplementary Information

Kinetic control over the chiral-selectivity in the formation of organometallic polymers on a Ag(110) surface

R.S. Koen Houtsma[1], Floris van Nyendaal[1], Meike Stöhr[1*]
[1] Zernike Institute for Advanced Materials, University of Groningen, Nijenborgh 4, 9747AG, Groningen, The Netherlands. E-mail: m.a.stohr@rug.nl

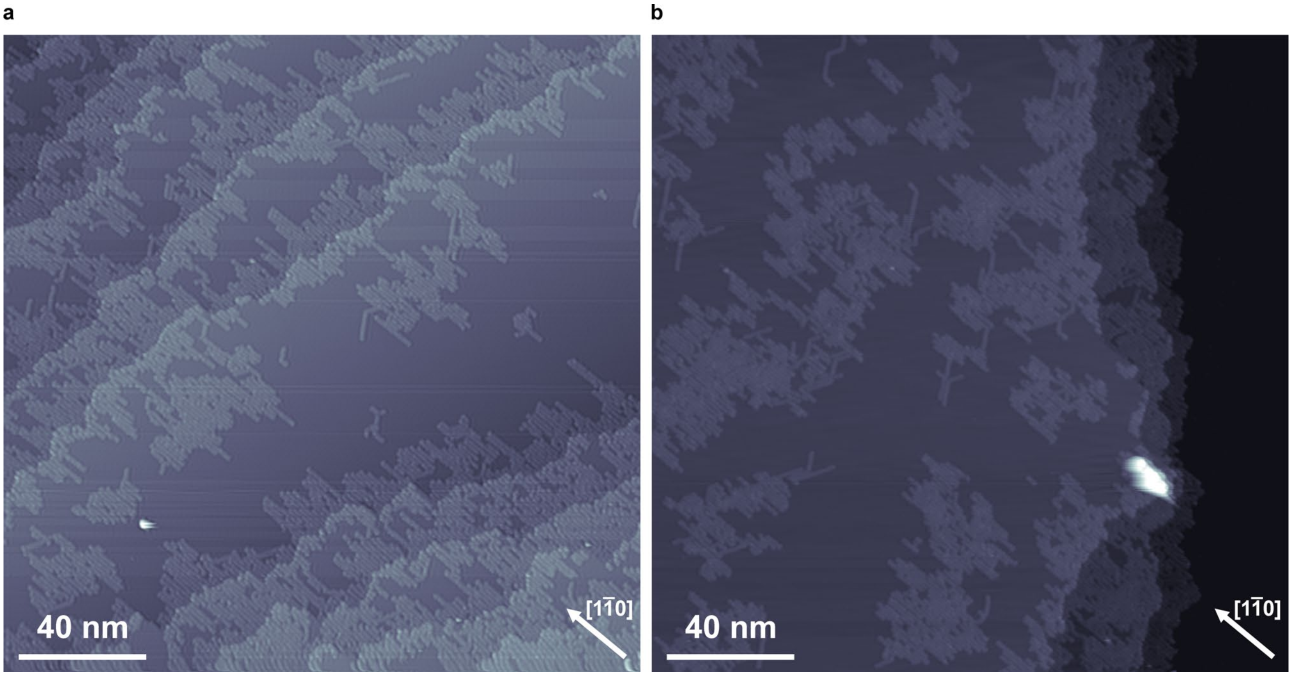

Figure S1. Overview STM images of monomer 1 deposited on a room temperature sample. The alignment of the islands with the $[1\bar{1}0]$ direction is visible in the images. Scanning parameters: a) -1.9 V, 30 pA. b) -1.9 V, 5 pA.

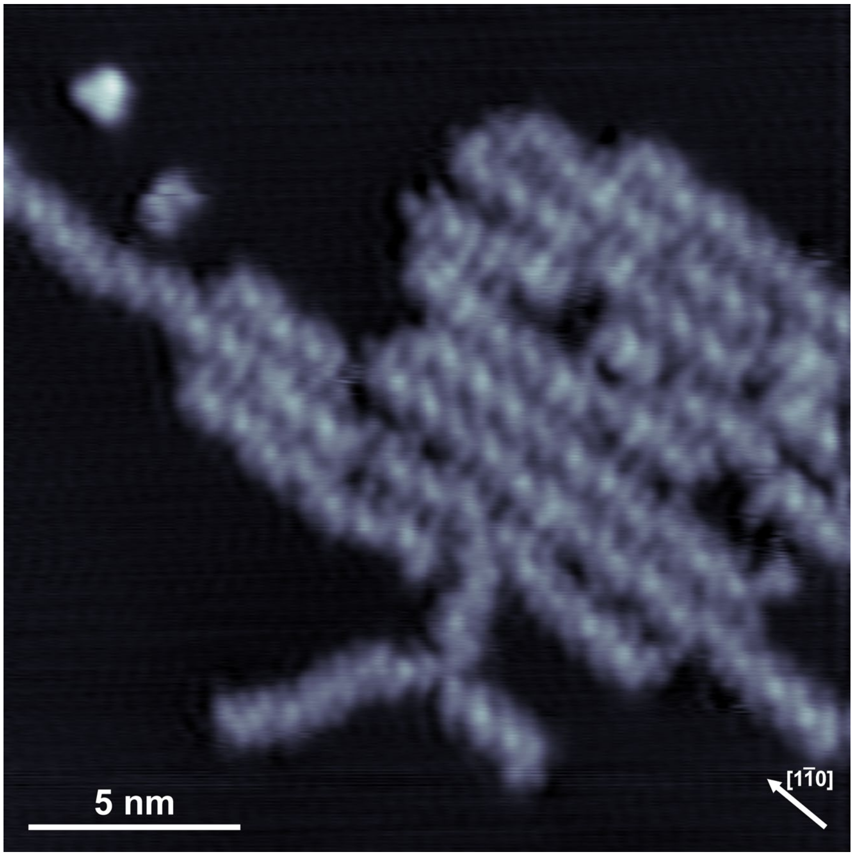

Figure S2. Close-up STM image of structure **2.** The internal structure of the 1D polymers is visible. Scanning parameters: 1.0 V, 100 pA.

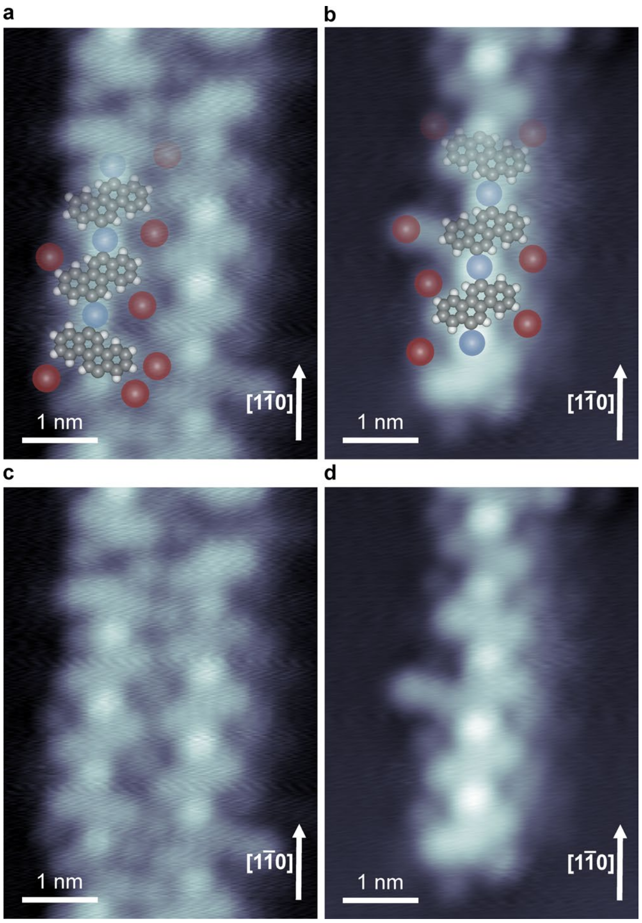

Figure S3. **STM images of heterochiral metal-organic polymers with and without overlaid tentative molecular model.** a,c) Two polymers side-by-side with (a) and without (c) overlaid tentative molecular model. b,d) A single polymer with (b) and without (d) overlaid tentative molecular model. Scanning parameters: a-d) U_{bias} = 0.5 V, I_{set} = 100 pA. Color code: C: gray; Br, red; Ag, blue; H, white.

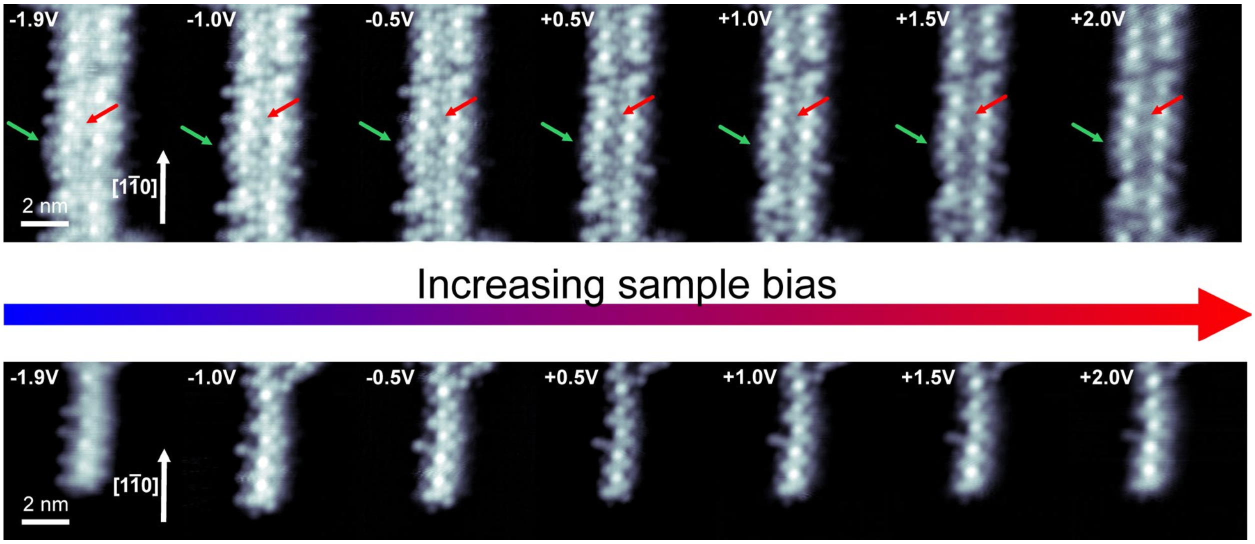

Figure S4. STM images of structure 2 at various biases. Top images show two polymers side-by-side. The red and green arrows point towards (the same) Br adatoms in all images. At sufficiently large sample bias (>1.5 V) the Br adatoms are invisible. Bottom images show an isolated polymer. Scanning parameters: V_{bias} given in figure, I_{set} = 100 pA for all images.

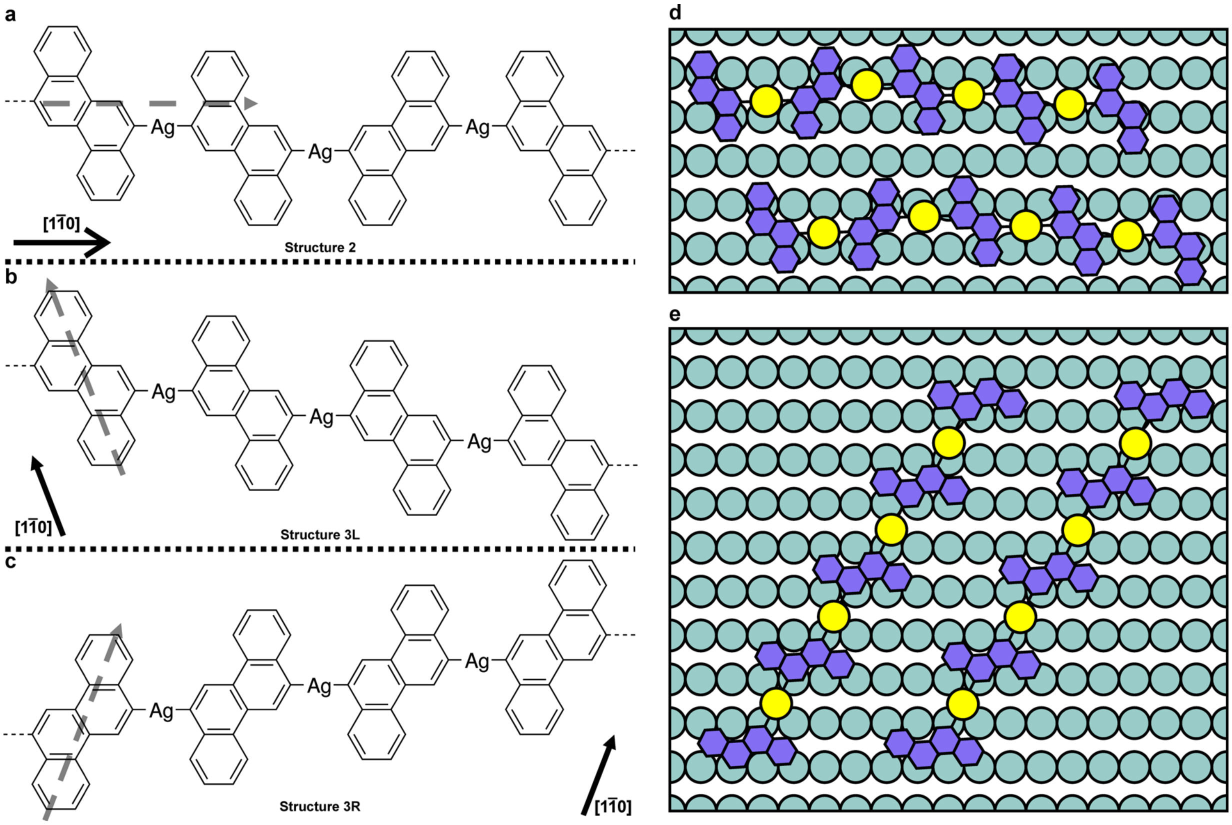

Figure S5. On-surface structures *versus* the $[1\bar{1}0]$ surface direction. a,b,c) Tentative molecular models of structures **2**, **3L**, **3R**, respectively. The angle at which the structures form with respect to the $[1\bar{1}0]$ surface direction was determined experimentally using STM. d,e) Tentative molecular models of structures **2** (d) and **3L** (e) on the Ag(110) substrate, based on the experimentally determined unit cell parameters and angles with respect to the substrate. Color code: Ag surface atoms: cyan; coordinating Ag atoms: yellow; chrysene: purple hexagons.

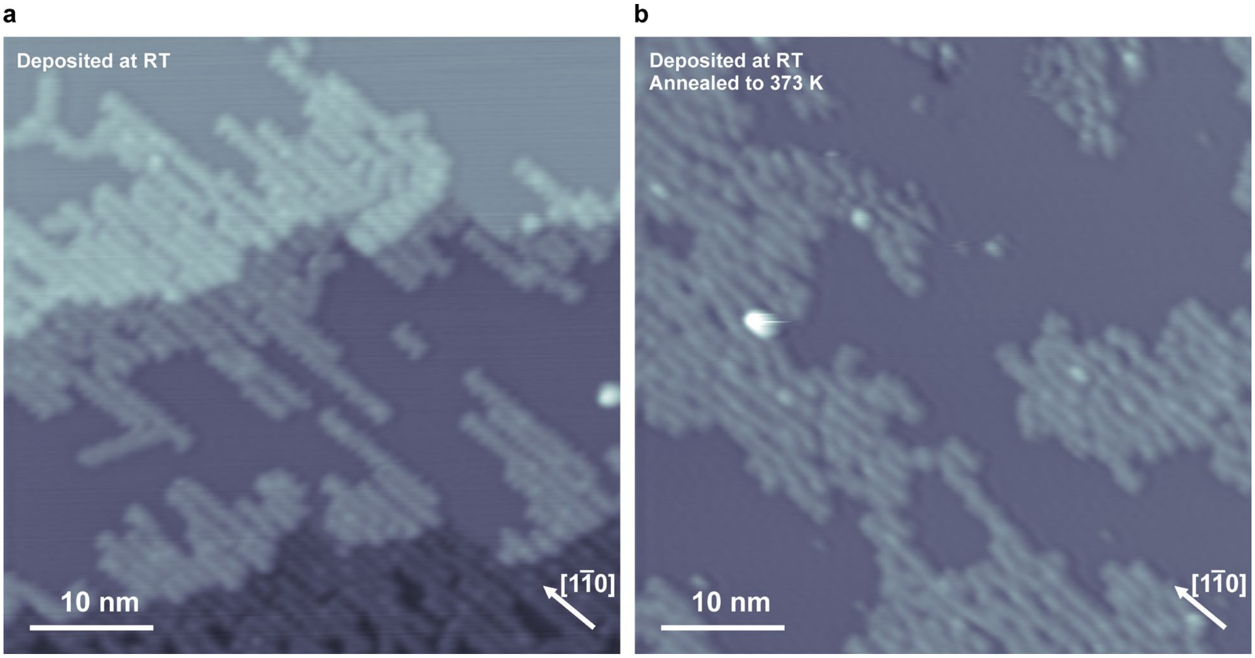

Figure S6. STM images of structure **2** before (a) and after (b) annealing at 373 K. The structure of the islands changed upon annealing, while the polymer length did not visibly change.

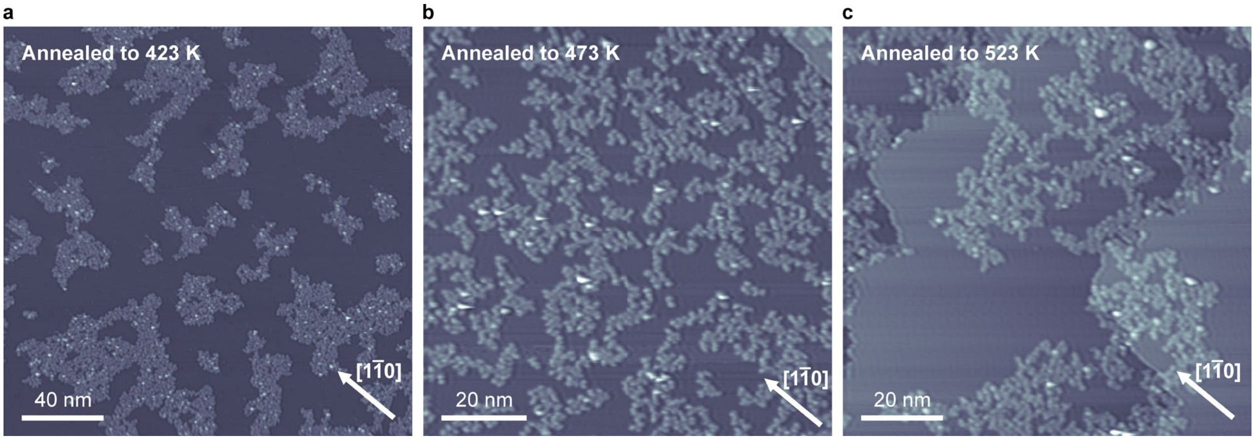

Figure S7. Overview STM images for annealing of room temperature deposited samples. a,b,c) STM image of a sample obtained by annealing the sample in Fig. 3a to 423 K. Small patches of disordered network are present. b) STM image of a sample obtained by further annealing the sample in (a) to 473 K. Patches of disordered, possibly partially polymerized networks are formed. c) STM image of a sample obtained by further annealing the sample in (b) to 523 K. Scanning parameters: a) -1.9 V, 5 pA, b,c) -1.9 V, 20 pA.

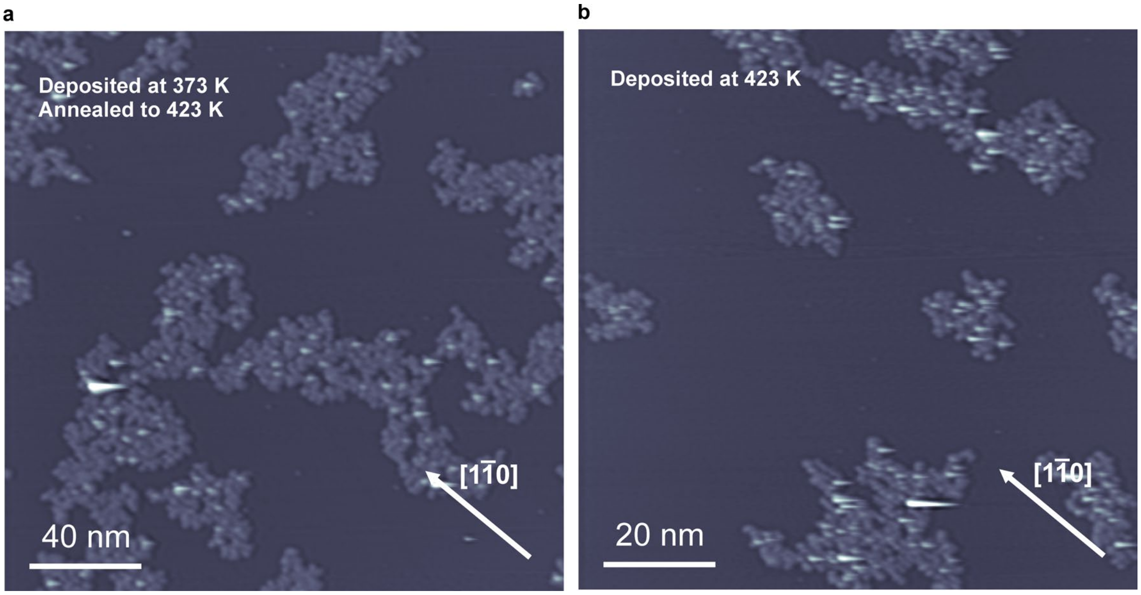

Figure S8. a) Overview STM image of the same sample as in Fig. 2a after annealing at 423K. b) Overview STM image of a sample deposited onto Ag(110) held at 423 K. a) -1.9V, 20 pA, b) -190 mV, 5 pA.

Supplementary Note 1

Annealing structures 3L and 3R

To investigate whether organometallic polymers **3L** and **3R** could be transformed into 1D covalently linked polymers or GNRs, the monomers **1** were deposited on Ag(110) held at 373 K and the sample was subsequently annealed at 423 K (Fig. S6a). This led to a similar disordered structure as was obtained when annealing a sample at 423 K which was prepared by depositing **1** onto Ag(110) held at RT.

Since it appears that the energy barrier to remove the Ag adatoms from the organometallic polymers is preventing the formation of covalently linked polymers, we deposited monomer **1** on a sample held at 423 K in an effort to promote the direct formation of C-C bonds, i.e. without the intermediate step of forming organometallic polymers. The results of this are shown in Fig. S6b. Again, disordered networks of monomer **1** were obtained. Thus, neither can the organometallic 1D polymers be transformed into 1D covalently linked polymers nor can the covalently linked polymers be obtained through a direct approach. This result that neither covalently linked polymers nor GNRs can be fabricated is similar to what was previously observed for the case of monomer **1** on Cu(111).[1]

A possible reason why no covalent coupling could be observed for the homo- as well as heterochiral polymers is that for both polymers covalent coupling between enantiomers of the same chirality would be required, which was reported to be energetically unfavorable.[1] In particular, transforming the organometallic polymers into covalently coupled ones would face steric hindrance between the monomers, which would require a (unfavourable) deplanarization of the monomers/organometallic polymer to alleviate this hindrance. Additional energy would be needed which would even further hamper the transformation from organometallic to covalently linked polymers.[2,3] Lastly, the split off bromine atoms that are co-adsorbed on the Ag surface and interact with the organometallic polymers via attractive H-bonding may hinder the formation of covalently coupled structures.

Supplementary References

1. Pham, T. A., Tran, B. V., Nguyen, M. T. & Stöhr, M. Chiral-Selective Formation of 1D Polymers Based on Ullmann-Type Coupling: The Role of the Metallic Substrate. *Small* **13**, 1–6 (2017)
2. Pham, T. A. *et al.* Comparing Ullmann Coupling on Noble Metal Surfaces: On-Surface Polymerization of 1,3,6,8-Tetrabromopyrene on Cu(111) and Au(111). *Chem. Eur. J.* **22**, 5937–5944 (2016).
3. Lackinger, M. Surface-assisted Ullmann coupling. *Chem. Commun.* **53**, 7872–7885 (2017)
